# Clinical and demographic features of pediatric burns in the eastern provinces of Turkey

**DOI:** 10.1186/1757-7241-19-6

**Published:** 2011-01-18

**Authors:** Albayrak Yavuz, Albayrak Ayse, Yıldız Abdullah, Aylu Belkiz

**Affiliations:** 1 Department of General Surgery and Burn Unit, Erzurum Region Education and Research Hospital, Erzurum, Turkey; 2 Department of Infectious Diseases and Clinical Microbiology, Erzurum Region Education and Research Hospital, Erzurum, Turkey; 3 Department of Pediatric Surgery, Sisli Etfal Education and Research Hospital, Istanbul,Turkey; 4 Department of General Surgery, Erzurum Region Education and Research Hospital, Erzurum, Turkey

## Abstract

**Background:**

The aim of this study is to perform a retrospective analysis of the causes of burns observed in children in the eastern provinces of Turkey.

**Method:**

In this study, patients were studied retrospectively with regard to their age, sex, cause of burns, seasonal variations, social and economic factors, length of hospital stay, burned body surface area, medical history, site of injury, and mortality.

**Results:**

A total of 125 patients undergoing inpatient treatment were male, (53.2%) and 110 were female (46.8%). The most common causes of burns in patients treated on an inpatient basis were scald burns (65.5%) and tandir burns (15.7%). The mean total body surface area of all the patients was 12.17+9.86%. When the patients were grouped according to tandir, cauldron, and others burn causes, a significant difference was seen between the in burn percentages caused by tandir and cauldron burns and other causes (p < 0.001). Higher burn percentages were seen for cauldron burns than for tandir burns (p < 0.05). The average length of hospital stay was 17.67+13.64 days. When the patients were grouped according to burn causes (tandir, cauldron, and others), a significant difference was determined between the hospitalization periods of patients with tandir burns and other burn causes (p = 0.001) The most commonly proliferating microorganism in burned areas was *Pseudomonas aeruginosa *(20.4%). Of the 235 patients, 61 were treated in operating rooms. During the 24-month period of the study, 2 of the 235 patients died (0.85%).

**Conclusion:**

Pediatric burns in the eastern part of Turkey are different from those in other parts of Turkey, as well as in other countries. Due to the lifestyle of the region, tandir and cauldron burns, which cause extensive burn areas and high morbidity, are frequently seen in children. Therefore, precautions and educational programs related to the use of tandirs and cauldrons are needed in this region.

## Background

Burns are serious health problems and are the most frequent injury among pediatric patients [1]. The incidence of burns, their treatment, and rehabilitation processes have a considerably marked effect on children in both physical and psychological terms [2]. Patients who recover from burns often will later have difficulties due to contractures, deformities, and functional limitations caused by scar tissue. Scar tissue treatment requires a prolonged period and also constitutes a heavy economic burden on families and the government [2].

Burns observed in patients under the age of 20 years are generally caused by accidents, many of which are avoidable. Severe burns are one of the major causes of morbidity and mortality in juveniles, and they are the third most frequent cause of deaths due to injuries in this age group [3]. Epidemiological studies of burn injuries have highlighted specific risk factors and have led to the establishment of effective preventive programs [4,5]. Programs for domestic accidents are estimated to prevent 70% of the deaths of children caused by burns [6]. When severe burns in children are treated aggressively in well-equipped burn treatment centers, good prognosis can be achieved [7-9]. The aim of this study was to perform a retrospective analysis of the causes of burns observed in children in the eastern provinces of Turkey, the available treatment methods, and complications that would typically arise.

## Methods

The population of the region served by the centers is 5,572,854, consisting of 2,845,920 males and 2,726,934 females. (The entire population of Turkey is 72,561,312, consisting of 36,462,470 males and 36,098,842 females) [10]. Our burn treatment center is located in one of the largest provinces in the Eastern Anatolia Region of Turkey, in Erzurum, and serves approximately 5,500,000 people, with patients also coming from nearby provinces. According to the population poll of TUIK, the child population is 21,484,334 in the whole of Turkey, and it is 1,837,216 in the study area [10].

In the 24-month period between September 2008 and September 2010, 545 patients presented at our hospital with different causes of burns. Since 310 of these patients had no indications for inpatient treatment, their treatments were carried out on an outpatient basis. The remaining 235 patients underwent inpatient treatments. The criteria for inpatient treatment was burns over 10% of total body surface area; burns on the face, hand, foot, perineum, or major joints; circular burns on extremities; full-thickness burns over 5% of the total body surface area; and electrical, chemical, and inhalation burns. An electronic database of medical records available at the burn center allowed all patients admitted to the center to be traced.

Patients were studied retrospectively with regard to their age, sex, cause of burns, seasonal variations, social and economic factors, length of hospital stay, burned body surface area, medical history, site of injury, and mortality. Patients were divided into 3 groups in terms of total affected body surface area (0-5, 15-30, and over 30%), the length of hospital stay (under 10 days, 10-20 days, and over 20 days) and the socioeconomic status (family earning less than 400 dollars per month, 400-800 dollars per month, and over 800 dollars per month). In addition, the patients were also grouped as to burn causes: tandir (clay oven), cauldron (cokelek), and other causes.

The treatment protocol was established in accordance with the main international standards of treatment and included resuscitative regimens, antibiotherapy, wound care, and surgical operations. Burn wound infection criteria were as detailed by the American Burn Association Consensus Conferences [11].

Data were analyzed by the Statistical Package for the Social Sciences, a commercially available statistics software package (SPSS, Chicago, IL). All data were presented as means (±) standard deviations (S.D.). Parametric tests were performed for data analysis. A one-way ANOVA test was performed and post-hoc multiple comparisons were done with least significant difference (Tukey). These differences were considered significant when probability was less than 0.05.

## Results

A total of 125 e patients who underwent inpatient treatment were male, (53.2%) and 110 were female (46.8%). When the patients were grouped according to burn causes (as tandir, cauldron, or other burns), no correlation was noted between burn causes and the age of the patients (p > 0.05).

Frequent cases of burns were observed during the summer, when tandir burns are quite common. Table 1 shows the monthly frequency of summer burns.

**Table 1:** Burn characteristics by total body surface area (TBSA) groups

	0-15%	15-30%	>30%	Total patients
Hospitalisation time	14.5 ± 11.5 day	23.8 ± 13.7 day	33.8 ± 17.5 day	235 (100%)
Etiology of burn injury				
Scalding	128 (54.5%)	29 (12.3%)	3 (1.3%)	160 (68.1%)
Tandir	25 (10.6%)	7 (3.0%)	5 (2.1%)	37 (15.7%)
Cokelek	6 (2.6%)	5 (2.1%)	6 (2.6%)	17 (7.3%)
Flame	8 (3.4%)	3 (1.3%)	2 (0.8%)	13 (5.5%)
Electrical	3 (1.3%)	2 (0.8%)	0 (0%)	5 (2.1%)
Contact	3 (1.3%)	0 (0%)	0 (0%)	3 (1.3%)
Monthly distribution of admissions to the hospital				
January	13 (5.6%)	3 (1.3%)	1 (0.4%)	17 (7.3%)
February	13 (5.6%)	4 (1.7%)	0 (0.4%)	17 (7.3%)
March	7 (3.0%)	3 (1.3%)	0 (0%)	10 (4.3%)
April	9 (3.8%)	2 (0.8%)	0 (0%)	11 (4.6%)
May	12 (5.1%)	7 (3.0%)	1 (0.4%)	20 (8.5%)
June	20 (8.5%)	3 (1.3%)	6 (2.6%)	29 (12.4%)
July	20 (8.5%)	9 (3.9%)	3 (1.3%)	32 (13.7%)
August	26 (11.1%)	3 (1.3%)	1 (0.4%)	30 (12.8%)
September	10 (4.3%)	2 (0.8%)	0 (0%)	12 (5.1%)
October	9 (3.8%)	5 (2.1%)	2 (0.8%)	16 (6.7%)
November	15 (6.4%)	4 (1.7%)	1 (0.4%)	20 (8.5%)
December	19 (8.0%)	1 (0.4%)	1 (0.4%)	21 (8.8%)
Socioeconomic status of patients family				
Group 1	48 (20.4%)	18 (7.7%)	12 (5.1%)	78 (33.2%)
Group 2	119 (50.6%)	26 (11.1%)	4 (1.7%)	149 (63.4%)
Group 3	6 (2.6%)	2 (0.8%)	0 (0%)	8 (3.4%)

Group 1: family earning less than 400 dollars per month, Group 2: family earning 400-800 dollars per month, Group 2: family earning over 800 dollars per month; TBSA: total body surface area

The most common causes of burns in patients treated on an inpatient basis were scald burns and tandir burns. Another frequently encountered cause of burns was cauldron burns, which occurred when children fell into cauldrons. Table 1 shows the causes of burns and socioeconomic status of the family.

A total of 123 patients treated on an inpatient basis in our burn treatment center were from Erzurum and its counties. The other 112 patients were referred from neighboring provinces. In all, 185 of the patients were from the countryside (78.7%) and 50 were from inner cities (21.3%).

The most common burn locations on patients were the front and back of the torso (trunk). For tandir burns especially, the upper and lower extremities were the most affected parts. Hands were common sites of electrical burns, while the front and back of the torso and the upper extremities were common sites of cauldron burns. The head and neck region were also among the most affected parts (Table 2 shows the number of patients and the parts affected by burns). When the patients were grouped according to burn causes (tandir, cauldron, or other causes), a significant difference in burn percentages was seen between the tandir and cauldron burns and the other causes (p < 0.001). Higher burn percentages were seen for cauldron burns than for tandir burns (p < 0.05).

**Table 2:** Anatomical sites, causative organisms and treatment in patients

	n	%
Anatomical sites		
Trunk	93	39.5
Leg	75	31.9
Arm and hand	69	29.4
Head	55	23.4
Major joint	64	27.2
Perineum	6	2.6
Causative organisms from patients		
Pseudomonas aeruginosa	48	20.4
MRSA^a^	19	8.1
Enterobacter	6	2.6
MRCNS^b^	5	2.1
E.Coli	4	1.7
Acinetobacter	2	0.9
Others	10	4.3
Treatment modality		
Debridment	61	26
Escarotomy	13	5.5
Fasciatomy	3	1.3
Grafting	58	24.7
Amputation	11	4.7

a: Methicilline Resistant Staphilococcus Aureus, b: Methicilline Resistant Coagulase Negative Staphilococcus

The mean total body surface area (TBSA) of all patients was 12.17+9.86% (Table 1 shows the characteristic features of TBSA groups). A significant difference was found for hospitalization periods of patients with 15-30% and over 30% burns compared with those with 0-15% burns (p < 0.001). The hospitalization periods of patients with 15-30% burns were less than those of patients with over 30% burns (p < 0.05).

The average length of hospital stay was 17.67+13.64 days. The longest hospital stay occurred with the patients with tandir burns, who had hospital stay durations of 26.6+12.3 days. When the patients were grouped according to burn causes (as tandir, cauldron, or other causes), a significant difference was determined between the hospitalization periods of patients with tandir burns and other burn causes (p = 0.001); however, no difference was noted for hospitalization duration between tandir and cauldron burns (p > 0.05). No difference was noted between cauldron burns and other burn causes (p > 0.05).

During the hospital stays of patients in our burn treatment center, the growth of wound cultures was observed in 94 patients. The most commonly proliferating microorganism was *Pseudomonas aeruginosa*. Bacillary Angiomatosis developed in one patient. The number of patients and their microorganism types are shown in Table 2. Proliferation was observed in the burn cultures of 28.3% of the patients with 0 - 15% burns, in 65.2% of the patients with 15 - 30% burns, and in 93.7% of the patients with over 30% burned surface area.

A total of 22 patients who came to our hospital had some type of substance applied to their burns, either toothpaste, potato, yoghurt, molasses (a type of jam made from hot grape juice), or shoe polish.

Although 227 patients had no serious health problems, 5 of the patients had epilepsy and 3 of them had mental retardation. Of the 235 patients, 61 were treated in operating rooms. The patient group that required the greatest number of surgical procedures consisted of patients with tandir burns. Table 2 shows the number of surgical procedures. Of these patients, 2 were connected to a mechanical ventilator, but died before tracheostomy could be performed.

During the 24-month period of the study, 2 of the 235 patients died (0.85%). One of these fatalities was a 7-year-old male with inhalation burns and 55% TBSA burns. The other was a 3-year-old female with 30% TBSA burns and who had started to develop gastroenteritis 3 days before the burn. In 6 patients whose TBSA was over 30%, thrombocytopenia due to sepsis developed. Since we did not have an opportunity to apply thrombocyte suspension to our patients, we referred these patients to another hospital, where thrombocyte suspension could be applied.

## Discussion

Patients accepted to our burn treatment center came from the province of Erzurum, where our treatment center is located, as well as from neighboring cities. Burn victims were more frequently males than females; the male prevalence (53.2%) was similar to that reported in other studies [12,13]. No tendency was noted for burn injuries to occur in the winter season. On the contrary, a trend was evident toward increased incidence of injuries in the summer season; we saw the highest number of admissions in June, July, and August. In another study carried out in Turkey, burn cases were reported to occur most frequently in the months of May, June, July, and August, in agreement with our study [14]. We are of the opinion that the underlying reason for this summer burn frequency is due to the summer use of tandirs and cauldrons. Another possible explanation might be that the months of June, July and August are the summer vacation period, when children are at home, rather than at school, and therefore burn injuries were more likely.

**Figure 1:**
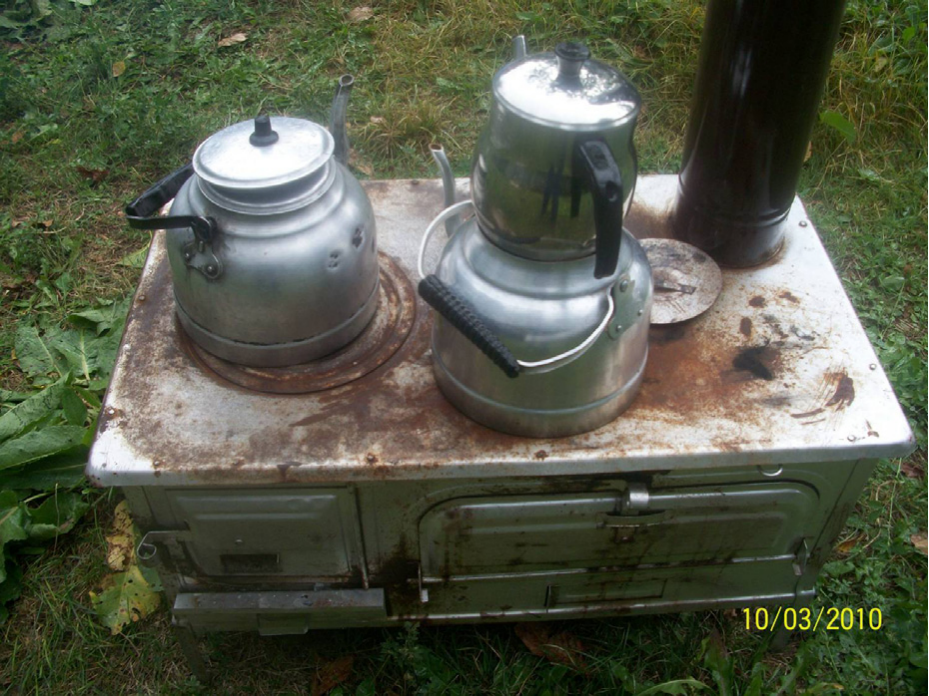
**Image of a stove heating oven used for heating and cooking**.

**Figure 2:**
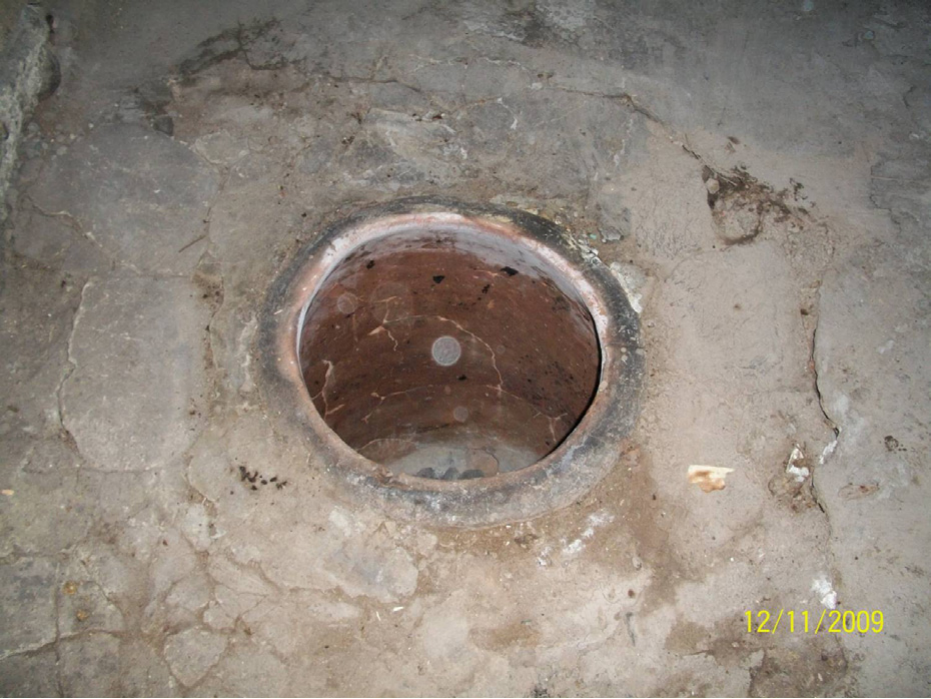
**Image of a tandir oven used for baking bread**.

**Figure 3:**
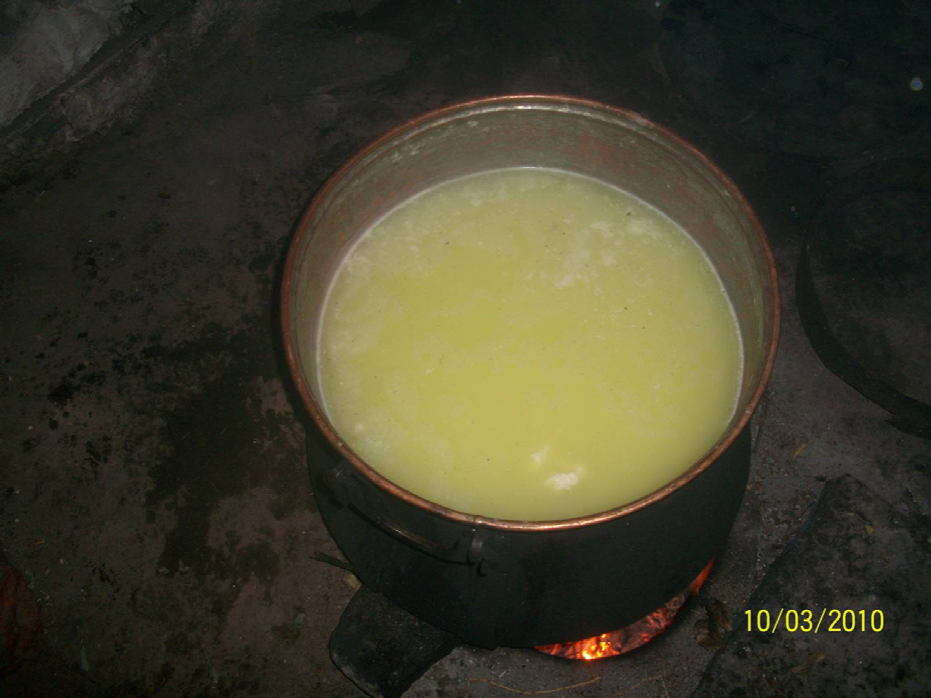
**Image of a cauldrons oven used for making cheese**.

Scalding was the most frequent cause of burns in this study. This is in line with some reports from other countries that describe high rates of scald burns in children of this age range [13,15-17]; however, the specifics seem to differ from region to region. In one study of children from Osaka, Japan, scalds that resulted from falling into large containers of hot liquid (bath scalds) were more frequent than non-bath scalds [13]. In contrast, investigations of children living in France and Iceland revealed that the incidence of non-bath scalding from hot liquids and drinks was higher than the incidence of bath scalds [18,19]. The region in which our treatment center is located is the region of Turkey with the lowest socioeconomic level and the area also has very cold winter days. A substantial number of people living in these provinces provide their heating and some cooking requirements with heaters referred to as "stove heating" (Figure 1). The use of these heaters in our region is the most important reason for scald burns. The second most important cause of burns is the tandir. A tandir is a buried oven used in bread baking in open air spaces (Figure 2). In a study on tandir burns, Akçay et al. indicated that 37 of 60 patients with tandir burns were children under the age of 10 [20]. Burns due to flames is another cause of burns in our province. In these cases, burns are generally caused by open fires. Rawlins et al indicated the rate for this type of burn as 11% [21]. Another cause of burns occurring in our province is burns due to cauldrons. In a major area of the Anatolian region, many people use deep copper and aluminum cauldrons to make their own traditional cheese, a so-called "dry cottage cheese" (Figure 3). Children can fall into these cauldrons, causing burns. Since the "dry cottage cheese" includes lactic acid, the burns that develop are also deep, which increases their morbidity [22].

Electrical burns generally develop due to children playing with sockets, while contact burns are usually due to touching a "heating stove." Harmel et al. noted that electrical burns were less common in the pediatric age group when compared with other types of burn injuries [23]. These results were also consistent with our findings. We found only 2.1% of the electrical burn injuries occurred in children. However, two studies published in Turkey by Haberal et al. [24] and Anlatici et al. [25] reported that the rate of electrical injuries in the pediatric age group was 10% and 16.8%, respectively. The reason for electrical burns being statistically higher in these two studies when compared to our study is due to the fact that burns due to tandirs and cauldrons are more frequently encountered in our province. Therefore, the rate of electrical burns in our study was relatively lower.

Low socioeconomic level also was an important factor in burn injury. Most of our patients were from the group with lowest income level. Coruh et al. [14] indicated in their study that 78% of pediatric burns occurred in people of low socioeconomic level, which paralleled with our findings. The most affected body parts in our study patients were the front and back of the torso, the feet, hands, head and neck region, major joints, and the genital region. In tandir burns, the hands, feet, and head and neck region were especially affected (Figure 1). Bekerecioglu et al. [26] treated 68 major burn cases in a 3-year period; 32 (47%) of these were due to tandir injuries. In their series, most of the burn victims were children and, in most of the cases, burn injuries included the head and both hands. These results were in agreement with the findings of our study. The length of hospital stay and the mortality increased with increases in extent of the burn area. In our case, the 2 patients who died had over 30% TBSA burns.

Epilepsy in 5 and mental retardation in 3 of our patients resulted in burns. The types of burns for these patients were contact and scald burns. In another study, 1 patient with psychiatric disease, 9 with epilepsy, and another with alcoholism were reported [14].

Two (0.85%) patients died from multiple organ failure due to severe sepsis. One of these children also had an inhalation burn. The TBSA of the patients who had died was over 30% and they had applied to our center from the countryside outside of our province. Higher mortality occurred among patients from rural areas than among those from urban environments, and this emphasizes the importance of factors such as inadequate first aid, poor transport conditions, and poor metabolic and hemodynamic resuscitation prior to reaching burn units. Previously, the mortality of pediatric burns has been reported to be 0.2-10.2% [27,28]. The rate of mortality in our study was in accordance with previous studies.

The average length of hospital stay was 17.67+13.64 days. The patients with the longest hospital stays were the patients with tandir burns. The average length of hospital stay in the study of Akçay et al. [20] was reported as 31.64 days, which was in accordance with our study. We have determined that patients with tandir and cauldron burns are hospitalized for longer periods than are patients with other types of burns. Burn surface percentage in cauldron burn victims was higher than for tandir or any other types of burns. We believe that the reason underlying the long hospital stays for patients with tandir burns is due to the fact that tandir accidents generally produce third degree burns in patients and these groups require operations like debridement, grafting, and amputations much more frequently.

Surgical procedures were applied to 61 patients in our burn treatment center. The types of surgical procedures were escharotomy, fasciotomy, escharectomy, debridement, split and/or full thickness skin grafting, and amputations. Escharotomy and fasciotomy were applied to patients with circular third degree burns to the extremities in case of a compartment syndrome, escharotomy or debridement was applied to patients with dense eschar, split- and full-thickness skin grafting was applied to patients whose burns were not epithelized within 3-4 weeks, and amputation procedures were performed on patients whose fingers and toes were totally burned and necrosed. Extremity amputation was not required for any patient in the present study. In the study of Akçay et al., 8 patients of 60 required an amputation procedure [20].

Since burns cause damage to the skin integrity, microorganisms can easily settle on the damaged skin. In our treatment center, infections developed due to a variety of microorganisms in the scars of 94 patients. The most prevalent microorganism with the most growth was *Pseudomonas aeruginosa*. Patients with scars in which microorganisms proliferated were evaluated by the Infectious Diseases Clinic and antimicrobial treatments were administered. In a study carried out by Ramakrishnan et al. on pediatric patients, *Staphylococcus aureus*, *Pseudomonas*, and *Klebsiella *were determined as the most commonly growing microorganisms on the scars of the patients [29]. In the study carried out by Akçay et al. [20], *Pseudomonas *(85%) and *Enterobacter aerogenes *(12%) were stated as the most rapidly growing microorganisms. The results of these studies were in agreement with our findings.

Under-resuscitation of a burn patient can lead to a downward spiral of unnecessary complications or to increased mortality [30]. To reduce morbidity and mortality, patients with any type of burns should be taken to a burn treatment center as soon as possible and their treatment should be started immediately. In our study, 2 ex-patients applied to our hospital 24 hour after burns occurred. Hagstrom et al. have reported that the prehospitalization fluid management of burn victims referred from outside emergency departments is inappropriate in 15% of patients [31].

Since the socioeconomic level of our province is low, some unusual traditional substances had been applied to some of our patients before they presented at our treatment center. Among the applied materials were toothpaste, potato, yoghurt, molasses, and shoe polish. In 4 patients, the color of the shoe polish was visible even when scars had epithelized, since the dye had penetrated deep into the inner layers of the skin.

## Conclusions

Burn cases cause permanent morbidities in many patients. Appropriate prehospital emergency care, taking the patient to the burn hospital as soon as possible, a well-equipped hospital with well-trained staff for burn treatment, well-timed referrals of patients, and appropriate treatment methods are important components of treatment of burn cases. Avoiding burn injuries is as important as treating patients with any type of burns to reduce morbidity. Pediatric burns in eastern Turkey are different from those in other parts of Turkey, and in other countries. Due to the lifestyle of the region, tandir and cauldron burns, which cause extensive burn areas and high morbidity, are frequently seen in children. Therefore, people living in these areas should be trained in socio-cultural terms and educated to recognize and avoid these burn dangers.

## Abbreviations

TBSA: mean total body surface area.

## Competing interests

The authors declare that they have no competing interests.

## Authors' contributions

YA and AA are the supervisor of the study., carried out control of and contributed to data extraction and writing of the study. AY and BA contributed to the data extraction. All authors read and approved the final manuscript.
